# Updating MISEV: When, What, How, and Why?

**DOI:** 10.1002/jev2.70230

**Published:** 2026-01-29

**Authors:** Naveed Akbar, Dylan Burger, Benedetta Bussolati, Edit I. Buzás, Randy P. Carney, Young‐Eun Cho, Tom A. P. Driedonks, Juan Manuel Falcon‐Perez, Yu Fujita, Deborah C I Goberdhan, An Hendrix, Yiyao Huang, Suresh Mathivanan, Mỹ G Mahoney, Sujata Mohanty, Andreas Möller, Nicole Noren Hooten, Stefano Pluchino, Carlos Salomon, Ana Claudia Torrecilhas, Sara I. Veiga, Kenneth W. Witwer

**Affiliations:** ^1^ Division of Cardiovascular Medicine, Radcliffe Department of Medicine University of Oxford Oxford UK; ^2^ Kidney Research Centre Ottawa Hospital Research Institute Ottawa Canada; ^3^ Department of Cellular and Molecular Medicine and School of Pharmaceutical Sciences University of Ottawa Ottawa Canada; ^4^ Department of Medical Sciences University of Turin Turin Italy; ^5^ Department of Genetics, Cell‐ and immunobiology Semmelweis University Budapest Hungary; ^6^ HUN‐REN SU Budapest Hungary; ^7^ Department of Biomedical Engineering University of California Davis USA; ^8^ Department of Food and Nutrition Andong National University Andong South Korea; ^9^ Department of CDL Research University Medical Centre Utrecht Utrecht The Netherlands; ^10^ Exosomes Laboratory, CIC bioGUNE‐BRTA, CIBERehd Bizkaia Spain; ^11^ Division of Next‐Generation Drug Development, Research Center for Medical Sciences The Jikei University School of Medicine Tokyo Japan; ^12^ Nuffield Department of Women's and Reproductive Health University of Oxford Oxford UK; ^13^ Laboratory of Experimental Cancer Research, Department of Human Structure and Repair Ghent University Ghent Belgium; ^14^ Cancer Research Institute Ghent Ghent Belgium; ^15^ Department of Laboratory Medicine Nanfang Hospital, Southern Medical University Guangzhou Guangdong China; ^16^ Department of Biochemistry and Chemistry, School of Agriculture, Biomedicine and Environment La Trobe University Bundoora Australia; ^17^ Research Centre for Extracellular Vesicles La Trobe University Bundoora Australia; ^18^ Departments of Pharmacology Physiology, and Cancer Biology Thomas Jefferson University Philadelphia USA; ^19^ Department of Otolaryngology‐Head and Neck Surgery Thomas Jefferson University Philadelphia USA; ^20^ Stem Cell Facility‐DBT Centre of Excellence for Stem Cell Research All India Institute of Medical Sciences New Delhi India; ^21^ Department of Otorhinolaryngology Faculty of Medicine The Chinese University of Hong Kong Shatin Hong Kong SAR; ^22^ JC STEM Lab of Personalised Cancer Medicine, Li Ka Shing Institute of Health Sciences The Chinese University of Hong Kong Shatin Hong Kong SAR; ^23^ Laboratory of Epidemiology and Population Sciences National Institute on Aging, National Institutes of Health Baltimore USA; ^24^ Department of Clinical Neurosciences and National Institute for Health Research (NIHR) Biomedical Research Centre University of Cambridge Cambridge UK; ^25^ Translational Extracellular Vesicles in Obstetrics and Gynae‐Oncology Group, University of Queensland Centre for Clinical Research, Faculty of Medicine The University of Queensland Brisbane Australia; ^26^ UQ Centre for Extracellular Vesicle Nanomedicine The University of Queensland Brisbane Australia; ^27^ Laboratório de Imunologia Celular e Bioquímica de Fungos e Protozoários, Departamento de Ciências Farmacêuticas, Instituto de Ciências Ambientais, Químicas e Farmacêuticas Universidade Federal de São Paulo (UNIFESP) São Paulo Brazil; ^28^ Krantz‐Family Center for Cancer Research Massachusetts General Hospital Boston USA; ^29^ Department of Medicine Harvard Medical School Boston USA; ^30^ The Broad Institute of MIT and Harvard Cambridge USA; ^31^ Department of Molecular and Comparative Pathobiology Johns Hopkins University School of Medicine Baltimore USA; ^32^ Department of Neurology Johns Hopkins University School of Medicine Baltimore USA; ^33^ The Richman Family Precision Medicine Center of Excellence in Alzheimer's Disease Johns Hopkins University School of Medicine, Johns Hopkins Medicine, and Johns Hopkins Bayview Medical Center Baltimore USA

**Keywords:** extracellular vesicles, guidelines, ISEV, MISEV, minimal information, position papers, reproducibility

## 1 Introduction

The Minimal Information for Studies of Extracellular Vesicles (MISEV) is an ongoing project of the International Society for Extracellular Vesicles (ISEV) that now spans more than a decade (Lötvall et al. 2014; Théry et al. 2018; Welsh et al. 2024). Developing from expert opinion to evidence‐based, crowd‐sourced guidance to the extracellular vesicle (EV) field, MISEV has been welcomed by the community, with its three iterations receiving more than 17,500 citations. Although MISEV is not a prescriptive, inflexible guideline, its recommendations are meant to increase reproducibility. An apparent positive influence of MISEV has been reported in the literature (Van Deun et al. 2017; Poupardin et al. 2024). Representing the international EV community, the ISEV Board has taken the lead in building the previous versions of the document (Witwer et al. 2017; Witwer et al. 2021, Welsh et al. 2024). At in‐person meetings in April and November of 2025, the Board deliberated on several key questions about the future of MISEV: Is an update needed? If so, when? Should MISEV continue in its current shape or take an entirely new form, such as a wiki, a knowledge database that can be edited continuously by users and perhaps tied to a chatbot for personalized output? If continuing as a published paper, should MISEV remain a single document, be split into several equal documents, or become a combination of one main paper with several ancillary family members? This editorial of the ISEV Board presents the content and outcomes of these conversations.

## 2 Is a MISEV Update Needed?

Just two years after the appearance of the last MISEV, it is fair to ask whether an update is needed. Some parts of the EV field change very little over many years, while others progress rapidly. Is it acceptable to prepare a new document in which some sections are largely unchanged? Board members reflected on the frequent (re)publication of certain clinical guidelines, which in some cases retain substantial content from previous, recent versions. The example of guidelines in the autophagy field was also raised, in which voluminous guidelines appear every four to five years (Klionsky et al. 2008). A rapid succession of publications, even if similar, could be seen as necessary to prevent a perception of outdatedness and consequent diminishment of engagement. On the other hand, the large investment that is necessary for a new document cannot be justified if the goal is simply to keep to an arbitrary timetable or to maximize citations. There must be a real need. Overall, the Board concluded that such a need exists and must be planned for: MISEV should continue to be updated as justified by progress in the field and the concomitant necessity for evolving guidance.

## 3 What is the Optimal Frequency for MISEV Updates?

Across three MISEVs, updates have taken progressively longer to write and publish. MISEV2014, an editorial of the ISEV Board, was published less than two months after Board deliberations on the initial draft. From MISEV2014 to the publication of MISEV2018, four years elapsed, including approximately one year of work (not including pre‐writing preparations) that was invested into MISEV2018. More than five years then went by before MISEV2023 appeared (accepted in December 2023 but published only in February 2024), and the writing and publication process took more than three years instead of one. In contrast with MISEV2018, which received a rapid editorial review, publication of MISEV2023 occurred only after one year of pre‐submission and post‐submission review (Welsh et al. 2024). The increased time between MISEVs is mirrored by an increase in authors, from 14 in 2014 to almost 400 in 2018 and more than 1000 authors for MISEV2023. The document itself has also grown considerably in length, topics covered, and detail. Together, these factors mean an increasingly larger effort requirement, likely translating into longer times, to coordinate increasingly longer updates.

What, then, is the optimal frequency for MISEV updates? The consensus of the ISEV Board discussion was that five to five and a half years is a reasonable target, assuming conditions like those around MISEV2023. This would place the appearance of the next MISEV as late as 2029. However, an earlier publication could be possible under different conditions. On content, for example, some Board members opined that several of the new sections in MISEV2023, such as guidance on specific EV sources or in vivo models, might best be spun out into separate, specific position papers or perspectives. These could be under a MISEV umbrella or not. A thereby simplified MISEV would be more consistent with the “minimal” specification and easier to develop. Similarly, after review by more than 1000 co‐authors and multiple rounds of review by the ISEV Board and other contributors, it might be possible to have a more rapid editorial review before publication, as in 2018. With simplified content and review processes, a new MISEV could appear earlier than 2029.

## 4 Paper or Database?

The discussion summarized above assumed that MISEV remains a traditional publication; other possibilities were raised, though, by the ISEV Board and in conversation with previous corresponding authors of MISEV. As a journal paper, any edition of MISEV is neither changing nor changeable after the publication date. Updates necessitate a new paper. Could MISEV take another form in the future? One option would be to develop MISEV as a database such as a wiki: an online document that can be edited in real time. Editing could be open to any EV community member or restricted to a group of authorized experts. Edits could appear immediately or only after review by senior editors. Proposed edits could also be posted in a comments feature for review by the community before incorporation. The versioning feature of a wiki database would mean that old versions could easily be retrieved or restored as needed, and the evolution of specific points could be traced. A wiki database could also be connected to a chatbot, allowing users to ask questions and receive tailored responses.

The wiki database option has advantages and disadvantages versus the traditional paper approach. A clear advantage is the nimbleness of responding to new developments and novel technologies. The process behind all updates and consensus‐building would also be transparent for a wiki with versioning and community comment functions. Disadvantages were also noted by the ISEV Board. Who would host and maintain the wiki/database, and with what funds? Highly active editors could skew content towards their own opinions or work, if review conditions allow. To prevent this and ensure quality, senior editors would have to commit substantial time to checking content and policing editing behaviour (Wikipedia administrators will be familiar with this disadvantage). Perhaps more importantly, the constant flux of the document would make interpretation and citation difficult for users. It would be less straightforward to track uptake and citations of a wiki page compared with a journal article. If a chatbot were to be integrated with the wiki or other database, its performance would also require constant monitoring. Overall, the ISEV board felt that the disadvantages of a database system greatly outweighed the advantages, and they decided that MISEV should remain a published paper for the foreseeable future.

## 5 To Split or Not to Split?

As noted above, some parts of the EV field age better than others. Ultracentrifugation and transmission electron microscopy have been with us for almost a century, and the basics do not change substantially. In contrast, other techniques, for example, single‐particle capture and characterization approaches, are developing rapidly. Meanwhile, various clinical applications of EVs are also evolving quickly. Should each MISEV strive to be a guide to all aspects of the field? Or should MISEV be split in some way to address different aspects at different times? On this point, members of the ISEV Board had a wide range of opinions and could be roughly divided between “splitters” and “lumpers.”

The “splitters” envisioned that a constellation of papers could grow up around MISEV, addressing areas that had changed or arisen in the last few years. Some suggested that there could be a line of stand‐alone “mini‐MISEVs” on broad areas, such as a MISEV on therapeutics (“MISEV‐T”), diagnosis (“MISEV‐D”), or characterization (“MISEV‐C”). Others saw an opportunity for smaller papers that are connected to MISEV but that extend or develop specific, granular areas, such as one EV source or one technique. These could be designated as “MISEV family papers” or “MISEV companion documents.” Development of highly specialized guidelines by a relatively small group of experts would still require vetting but would avoid time‐consuming integration into a larger whole of a single MISEV document, expeditiously bringing information and recommendations to the community. Another advantage of splitting could be giving greater agency to subfields that have not received strong coverage in past MISEVs, such as non‐mammalian EV sources.

In contrast, the “lumpers” felt strongly that one of MISEV's greatest strengths is its coverage of so much of the EV field as a single document, albeit divided into chapters. MISEV is seen by many as a “one‐stop‐shop” for all things EV. Furthermore, MISEV has strong community buy‐in and respect in part because so many community members were involved in its creation. Fragmenting MISEV would result in papers with fewer authors and only partly overlapping authorship. A side‐effect could be loss of consistent quality. Another disadvantage would be diminishing the influence of the MISEV “brand” by placing the name on products that may not truly be “minimal information” recommendations. Additionally, despite possible efficiency gains by restriction to individual topics, central coordination would still be required, and the logistics would be greater than for one central document. In the view of the lumpers, breaking MISEV into smaller pieces would have a dilution effect on the MISEV brand and on its influence.

Despite the seeming incompatibility of the splitting and lumping approaches, a possible compromise was also floated, and from the April and November 2025 Board meetings, a strong consensus built around this approach: MISEV should remain a central document with regular updates. More peripheral topics, including some that were addressed partially in MISEV2023, could still be tackled by centrally coordinated but separate papers that would not carry the MISEV name but would be categorized instead as ISEV position papers or other ISEV products. Following this middle ground approach would have two main results. First, it would allow MISEV to be renewed without growing to an unwieldy length. Second, it would bring back the ISEV position paper as an important mainstay of the society's influence on the field. Early position papers of ISEV were well received and influential (Erdbrügger et al. 2021; Russell et al. 2019; Mateescu et al. 2017; Lener et al. 2015; Hill et al. 2013; Witwer et al. 2013), but no non‐MISEV position papers have been published in more than four years. The ISEV Board would like to facilitate a new blooming of position papers and other statements, whether from the whole society, the ISEV Board, or ISEV subgroups such as committees, task forces, special interest groups, and inter‐society working groups, with a path to ISEV endorsement and publication in ISEV journals.

## 6 Conclusions and Next Steps

After two in‐person ISEV Board meetings in 2025, and with conversations on and beyond the ISEV Board occurring during the intervening six months, several decisions were reached amongst a diversity of opinions. As outlined above, a new MISEV:

- is thought to be needed and will be prepared if confirmed by community input;
- should be published five years from the last MISEV publication if not earlier;
- will remain a traditional published document for now;
- will likely be simplified versus the MISEV2023 version to focus on minimal information rather than comprehensive reviews of the field and sub‐fields; and
- will leave room for new, MISEV‐related or ‐unrelated products to be commissioned and endorsed by ISEV to address important but relatively niche areas.

In addition, the ISEV Board resolved that the process of preparing the next MISEV update as well as other society products will engage the community as fully as possible, as was the case for MISEV2018 and MISEV2023. Whereas previous position papers other than MISEV usually arose from small but important ISEV Workshops, future position papers and similar products will be driven by needs identified by the community. These smaller products will arise through a process informed by the Delphi technique, with modifications as needed. The Delphi technique is a research methodology to build consensus among experts on matters characterized by some degree of uncertainty. In general, a facilitator collects expert opinions, summarizes the group's responses, and shares them back to the panel anonymously. Each round allows experts to revise their opinions based on the group's feedback, guiding them toward a collective judgment. (For a review of the evolution and different uses of the technique, see (Hasson et al. 2025).) More detailed guidance will be released in due course. Meanwhile, the ISEV Board has decided that the process will begin with an initial survey of the community for their help in identifying needs for MISEV, gathering assistance for its preparation, and pinpointing urgent topics for products other than MISEV.

## Funding

The authors have nothing to report.

## Conflicts of Interest

SP is founder, CSO and shareholder (>5%) of CITC Ltd; KWW consults as Kenneth Witwer Consulting.

## Preparation and authorship

This piece summarizes discussions at the ISEV Board meetings 1) in April 2025 at the ISEV2025 annual meeting in Vienna, Austria, and 2) in November 2025 preceding the ISEV EVs in Immunity meeting in Athens, Greece. An initial draft was written by KWW. Other members of the ISEV Board reviewed and revised the draft and are listed in alphabetical order.

## Data Availability

Data sharing not applicable to this article as no datasets were generated or analysed during the current study.
